# WeiBI (web-based platform): Enriching integrated interaction network with increased coverage and functional proteins from genome-wide experimental OMICS data

**DOI:** 10.1038/s41598-020-62508-8

**Published:** 2020-03-27

**Authors:** Aman Chandra Kaushik, Aamir Mehmood, Xiaofeng Dai, Dong-Qing Wei

**Affiliations:** 1 Wuxi School of Medicine, Jiangnan University, Wuxi, China; 2 State Key Laboratory of Microbial Metabolism and School of Life Sciences and Biotechnology, Shanghai Jiao Tong University, Shanghai, 200240 China

**Keywords:** Data integration, Data mining, Data processing, Genetic databases, Gene regulatory networks

## Abstract

Many molecular system biology approaches recognize various interactions and functional associations of proteins that occur in cellular processing. Further understanding of the characterization technique reveals noteworthy information. These types of known and predicted interactions, gained through multiple resources, are thought to be important for experimental data to satisfy comprehensive and quality needs. The current work proposes the “WeiBI (WeiBiologicalInteractions)” database that clarifies direct and indirect partnerships associated with biological interactions. This database contains information concerning protein’s functional partnerships and interactions along with their integration into a statistical model that can be computationally predicted for humans. This novel approach in WeiBI version 1.0 collects information using an improved algorithm by transferring interactions between more than 115570 entries, allowing statistical analysis with the automated background for the given inputs for functional enrichment. This approach also allows the input of an entity’s list from a database along with the visualization of subsets as an interaction network and successful performance of the enrichment analysis for a gene set. This wisely improved algorithm is user-friendly, and its accessibility and higher accuracy make it the best database for exploring interactions among genomes’ network and reflects the importance of this study. The proposed server “WeiBI” is accessible at http://weislab.com/WeiDOCK/?page=PKPD.

## Introduction

In the context of interactions, a brief explanation of the function and all functional interactions can be used to accurately narrow down a large amount of data. Having sufficient knowledge about interactions is a prerequisite as it reveals a dimensional view of many potential functional activities. Consequently, the complete description of biological phenomenon directly designates the specific interaction between entities^1–3^. For large assemblies of entities, a three-dimensional view can be more meaningful.

Cellular modes may be determined by mass transport while the sequestration of signaling interactions and molecular actions may be regulated as well by “cooperative binding”. Based on the valuable insights of interactions, notes have been added that categorize interacting proteins into functional sets that are labeled similar to signaling pathways, physical complexes and a limited tightly linked ‘modules’^4–6^. Nevertheless, the distribution of interactions into diverse complexes or pathways are divisible which are likely to prevent verification of the likelihood of crosstalk and dynamic states in the interacting domain^7^. One commonly employed approach is to avoid the subdividing of functions in a network, particularly creating a network that is based on topological outcomes of all types of known or predicted interactions. In the context of the network, a web-based system is considered outstanding when it accurately integrates numerous kinds of interactions that express stable physical partnerships, frequent attachment, chaining of a substrate, communication of data, and many others. The primary interaction repository^8–12^ provides an organized experimental dataset that includes multiple genetic, biochemical and biophysical techniques^13,14^. Progressions have focused on biological interactions from predicted computational data that are mainly focused on several forecasted communications using numerous algorithms^15–19^. Furthermore, the prospect of comprehensive and detailed coverage was elucidated using couple of web-based means that offers information about the combination of identified and forecasted communications. These databases mainly include STRING, GeneMANIA^20^, FunCoup^17^, I2D^21^, ConsensusPathDB^22^ and others that are based on specific necessities. The most flexible and stable online platform is the STRING database, which has allowed for confidential interactions, valuable scoring and detailed comprehensive analysis for many years. The primary interaction unit that is typically used for a specific and productive functional relationship regarding a protein interaction is a functional connotation. Interactions can be derived from various available sources, similar to known experimental interactions, counting primary databases, pathway data parsed within manually curated databases, automated text-mining for statistical or semantic connections in proteins, genomic and coexpression interactions’ analysis predicted *de novo*, and precomputed orthologs. Additionally, the interactions observed in one organism can be orderly transferred to another organism^22–24^.

The proposed WeiBI database predominantly focuses on gene (protein-yielding) alternative-loci splice isoforms or genes that are altered at the post-translational stage; further alterations are not available but are collapsed for a gene locus. The highly ranked functional grouping familiarized through unautomated curated Kyoto Encyclopedia^4,25,26^ of genes and genomes pathway maps provide the sources of interactions, and their declarations have been proven ideal. As stated earlier, WeiBI covers 115570 entries. To gain more knowledge of the biological phenomena, there are supplementary updates available for all the primary data resources, and aims to re-execute the text-mining pipeline with new and long technologies. Through extensive literature investigation, we examined many features and interfaces in other databases^27–29^. However, the data are not sufficient to be heavily banking on. Hence, we support ongoing studies that are focused on modifications and alternative additions to the database.

## Materials and Methods

### Implementation

The development of a particular tool or database requires the gathering of data and the implementation of various steps to efficiently process the existing data and flawlessly perform operations. The implementation methods may directly affect the reliability score and validity of the tool being developed. Thus, it is extremely central to examine the potentially available procedures and how they correlate with the designed model. In our proposed work, the methodological stages passed through during the development of WeiBI are discussed in depth in the next sections.

### Data mining for WeiBI

Obtaining relevant data and making it useful is a complicated task, but it also plays a crucial role in overall training and development, which may directly affect the validity of the model. Several databases were used to gather preprocessed information and data for WeiBI. Most databases for exploring interactions are molecular groups of precise information schemes that collect heterogeneous records of interactions formed between various entities.

### Data integration

WeiBI is a well-defined representation of coregulation as well as a strong indicator for functional associations. On the basis of coexpression, WeiBI version 1.0 is now planned with an enhanced and novel pipeline using all the available experimental microarray gene expression data provided by the Gene Expression Omnibus powered by NCBI (http://ncbi.nlm.nih.gov/). According to our current survey (March 2019), GEO (Gene expression Omnibus) includes more than 12,000 platforms (GPL), nearly 45,000 trials (GSE) and approximately 1 million matrices (GSM). Any amount of fragmented information in any analysis can create intricate circumstances. Consequently, the less-edifying matrices and platforms from individual trials are reduced by the tally of numerous miscellaneous arrangements in the breakdown. Primarily, studies showed that humans were grouped with specific data.

### Module access

**Figure 1:**
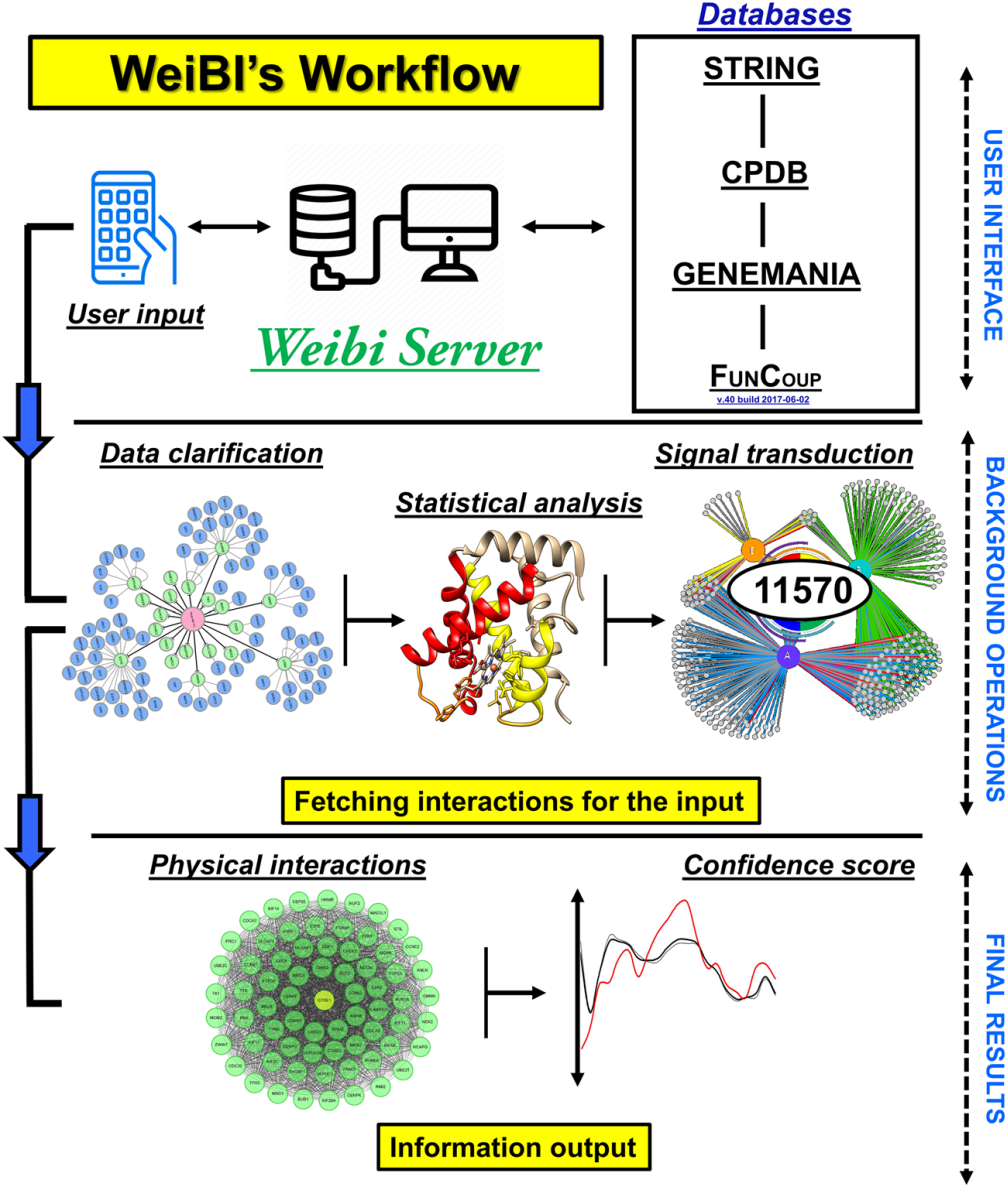
(Wei) The WeiBI workflow. Flowchart of WeiBI database accessibility and prediction through a GUI interface and automated background operations.

We have an alternative method to access the WeiBI database which is based on the application programming interface (API). WeiBI version 1.0 is directly accessible through WeiDOCK (http://weislab.com/WeiDOCK/) with the PHP programming environment. The PHP controls the standard WeiBI database (Fig. 1). This package is linked to the WeiBI server through an API and other additional web services such as (http://weislab.com/WeiDOCK/?page=Marvel). For proteomics data, information can be downloaded through the server which can be locally stashed in the PHP environment for all types of interaction networks, allowing the optimized subsequent accessions and data connotations. Apart from this, the package was designed around a graph framework that enables network information structure complexity and quickens the functional analysis. The key objective of the graph framework is that whenever we load a module into the backend control, the network can deliver high-level functioning, for instance, naming the mapped entities with their equivalent identifiers, recapturing the interested neighbor entities, and retrieving and stabilizing the link generation to support the WeiBI catalog. For a native representation, a plotted network is used that connects the entities via PHP scripting. To highlight and tag subsets of entities, we have existing functions for the augmentation of a specific network with available node colorings. We have performed statistical enrichment analysis on the listed genes inside the WeiBI namespace, including ontology of a gene, annotations regarding a pathway, and tissue/disease annotations. The visualization package provides lists of large entity sets that can deliver valuable insights and an online database interface, which has been proved to be a key contribution.

## Results and Discussions

### User interface

**Figure 2:**
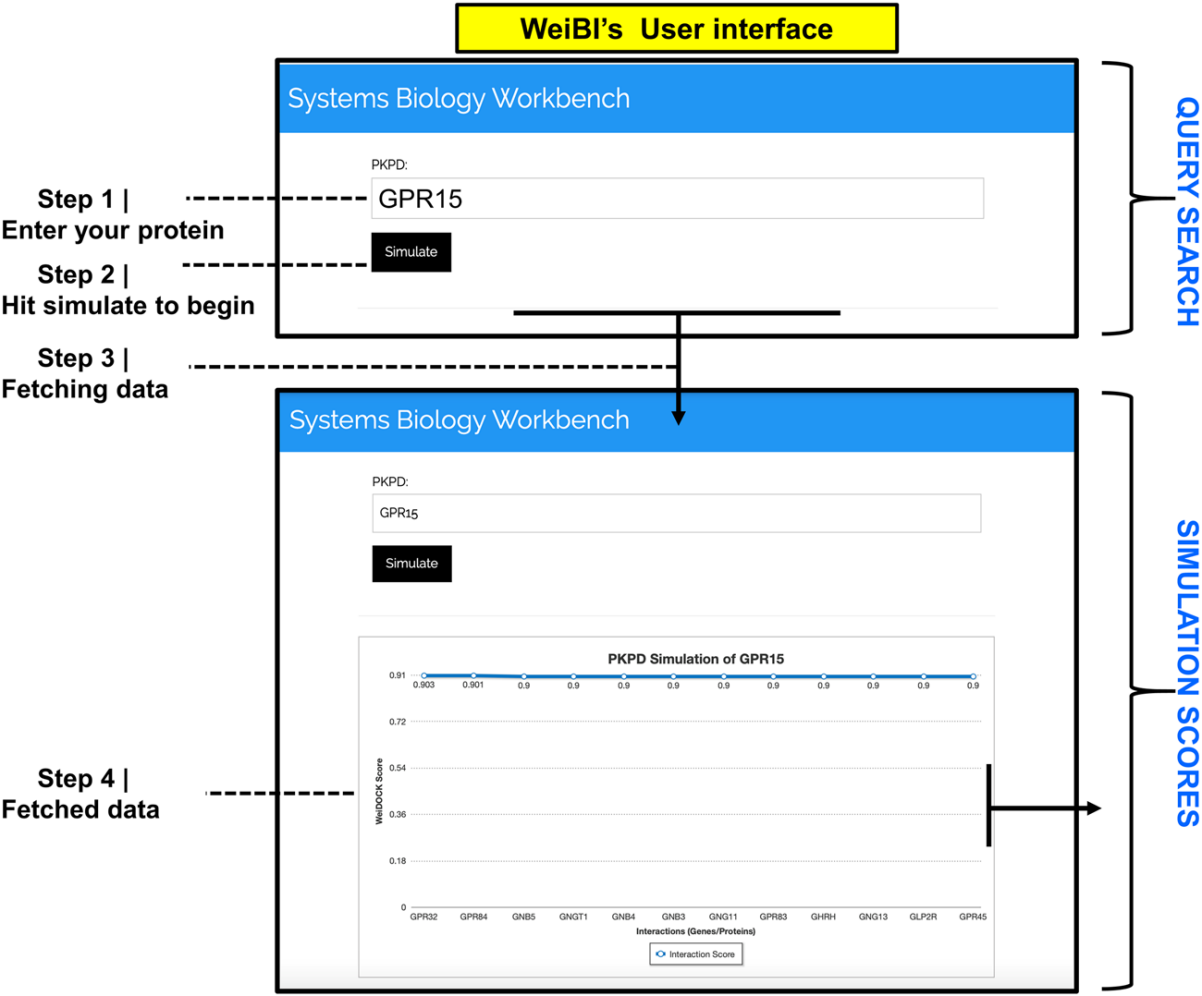
(Wei) The WeiBI database user interface. “GPR15” is queried in the search panel. Physical interactions and the confidence score are predicted by the WeiBI algorithm. A description is given for each component in a stepwise manner. Upon loading the webpage, a ‘search box is provided that can fetch information from a user’s query regarding a specific biological name or keyword such as “GPR15” as shown in the provided screenshot (Fig. 2). An alternative way to explore WeiBI is to simply select the “Input” option that generally allows us to upload the genes’ list and build identifier maps. This will show the data alongside interactions and any other related information. The user will first go through the identification process for individual entities or sets of inputs and then move to the network analysis. It is now possible to collect detailed information for interacting entities by reshuffling the score cutoffs and limiting the network size. By upgrading to the latest model, it is now possible to perform clustering, rearrangements, as well as statistical enrichment in the network.

The user interface of WeiBI covers all the available options incorporated in the database. All of the information provided by the WeiBI database website is designed to cover its various components. A description is given for each component in a stepwise manner. Upon loading the webpage, a ‘search box is provided that can fetch data from a user’s query regarding a specific biological name or keyword such as “GPR15” as shown in the provided screenshot (Fig. 2). However, for specific searches, we only retrieve restricted results that may include a weighted scheme both for the identifier and rank annotation text matches. The database can potentially be linked to other websites that crosslink with the WeiDOCK database, evolving the partner resources, such as Tool for Chemicals, and effectively share information concerning biological interactions, annotations and name-spaces with this database. An alternative way to explore WeiBI is to simply select the “Input” option that generally allows us to upload the genes’ list and build identifier maps. This will show the data alongside interactions and any other related information. The user will first go through the detection course for individual entities or sets of inputs and then transfer to the network investigation. It is now possible to gather comprehensive information for interacting entities by reshuffling the score cutoffs and restricting the network size. By upgrading to the latest model, it is now possible to perform clustering, rearrangements, as well as statistical enrichment in the network. WeiBI already performs enrichment detection, which covers associations with human diseases and tissue annotations. For these functions, the database connects with partner databases, sharing sequences and namespaces. This annotates entities to tissues or diseases, specifically through a mixture of computerized text-search and imported information.

### Interaction transfer between entities

Among the effective resources for organisms, WeiBI version 1.0 offers information about interactions and also transfer interaction’s knowledge from orthologous entities analyzed as organism interactions. Another fashionable feature of this version is its linkage to the precomputed orthologous relationships that are transferred through the eggNOG database, so-called ‘interlock’ transfer process. Orthologous in eggnog^30^ is a well-ordered pattern that supports the transfer of interactions in a crossing up and down manner. Preferably, the ingrained orthology should be self-disciplined, meaning the protein provided to the orthologous group for a particular phylogenetic array or higher-level arrays has to be well-classified. This is not always the case because of technical reasons (assigned orthologues are likely to be computed independently for every single array) shown in previous orthologous group versions., However, WeiBI version 1.0 contains a post-handling channel that successfully forms the entire arrangement not self-contradictory. Till we were able to make this feature gradually consistent, the database unceasingly implemented an iterative postprocessing pipeline on various levels. In the future, more consistent and patterned sets of genes, proteins’ families and orthologs will be used, enhancing the informative features in the user’s interface.

### Validation of WeiBI

**Figure 3:**
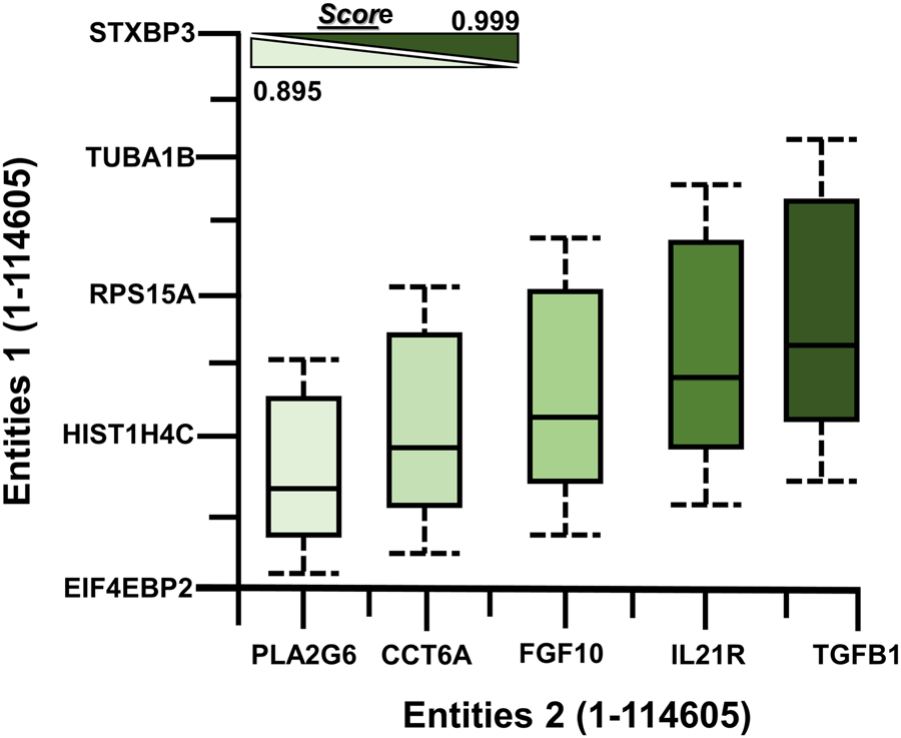
(Wei) WeiBI validation. Combined screenshots from WeiBI database predictions that have been queried with a subset of entities belonging to different complexes in humans. Boxplot peaks between the entities indicate interaction evidence for each protein. The color transition from light to dark green represents an elevation in the prediction score. This means that the light green box plot shows less value while the dark green color represents the highest prediction score. Additional information is available along with annotations (Additional file Table S1 where we did manual prediction from WeiBI to calculate accuracy and performance of WeiBI web-server).

Validation is a vital step that cannot be overlooked in the design of any tool, drug or application whose consequences are to be trusted upon (Fig. 3). For instance, in the generation of a pharmacophore model for drug discovery or a statistical model for protein communications, the models are first tested with substantiated data to measure the maximum precision before the model is introduced to the users. The current work tested 14605 entities for estimation purposes and achieved 90% of the accuracy as shown in the given figure. The user interface is also of prodigious importance. The simplicity and efficiency of a tool are crucial for attracting and allowing users to use the software for their purpose. The proposed database interface is easy to operate. The desired options can be selected on the server homepage, such as docking, pathway modeling or entity’s interactions. The homepage takes a user to a search box option where a valid keyword of interest can be entered that will subsequently fetch data at an optimized speed. Apart from the huge amount of GEO data used in the development of this platform, this database will keep on updating after particular intervals, such as after one year. Protein-protein docking is of great importance, and we aim to implement this function in our current server in the future. We are also looking forward to incorporating Genomics and Proteomics correlations, molecular interactions, and bond or distance calculations. This will require more improvements and flexibility in the existing algorithm along with mining and testing more and more data.

### Comparative analysis and spare features of WeiBI

WeiBI is a catalogue of various communications having information amassed by means of wide-ranging organizing exertions. Some of the main reasons why we developed WeiBI are mentioned below.

(i) WeiBI predicts direct and indirect partnerships associated with biological interactions. This database contains information regarding protein functional partnerships and interactions along with their integration with a computational prediction for humans and collects information using an improved algorithm by transferring interactions between more than 115570 entries, allowing statistical analysis with the automated background of given inputs for functional enrichment.
(ii) This approach also allows the input of an entity list from a database, the visualization of subsets as an interaction network and successfully performs enrichment analysis for a gene set.
(iii) We also believe there must exist a choice in the selection of a tool that can be helpful in a more accurate prediction by comparing the results of the same analysis performed by various tools.
(iv) If equated with databases like “STRING”, our database contains more data which can be confirmed by typing DEC2 and GPR7 genes for humans. Additionally, WeiBI’s algorithm has been highly improved (Here all-inclusive improvements in functionality and workability are offered based on wide-ranging usability checkups and employing the latest expertise, determined to provide a better experience to the user).
(v) This wisely improved algorithm is easy to use, and its accessibility and higher accuracy make it the best database for exploring interactions among network genomes, reflecting the importance of this study. For coping with this, the WeiBI (Wei for Biological Interactions’) is designed that assimilates these dissimilar information springs for 115570 entries into a solo, and user-friendly easy-to-use source. Besides the elevated demand of the repository, a novel grid sight is incorporated, offering the operator, the facility of exploring attachment affinities of the communications web.
(vi) WeiBI attains constantly improved functionality, fetching, and delivering of 97% of the illustrations under 1 nano-second. Merging the multi-filmed canvas approach of HTML5 and PHP along a space separating information building reduces the CPU’s stress, allowing novel features to be introduced. By means of extremely improved algorithms and data buildings, WeiBI has increased its functionality and conviviality regarding the fresh pathway illustration observer, that has made it more robust, accessible and easy-to-implement resolution. Since the statistical imagining of multifaceted information is a problem in computational biology, most of the distinct tactics offered here are applicable to an extensive array of online bioinformatics sources.
(vii) WeiBI server have some additional modules for users for additional analysis like- BCD: (http://weislab.com/bcd/index.php); JTDOCK: (http://www.weislab.com/WeiDOCK/index.php); WeiDOCK: (http://weislab.com/WeiDOCK/?page=Marvel); A-CaMP: (http://weislab.com/WeiDOCK/include/content/A-CaMP/A-CaMP.php); DTI: (http://weislab.com/WeiDOCK/index.php?page=DTI).

## Conclusion

The entire cellular mechanisms run under a highly controlled coordination system of various entities that interact with one another and the transduction of cellular signals happens. However, understanding this coordination system is extremely challenging. This formation of chemical interactions among various biological entities and the techniques used to analyze these linkages are crucial to understanding complex biological cascades. Different on/offline servers and tools are available to recognize interactions formed between or among the proposed entities, e.g., gene-gene or protein-protein interactions. This information can be used to design various therapeutic strategies to overcome a biological target, though it significantly hangs on on the precision and value of the predicted data. Thus, there exists a demand for available and accurate interaction information, signifying why this is one of the main challenges in molecular systems biology. Here, a novel database named as WeiBI (Version 1.0) is introduced that identifies interactions’ networks in the genome. WeiBI has been deliberate designed with unique attention to quality and control for interpreting direct or indirect biological networks created independently by specifically associated cohorts. The data used for the development of this database has been derived from numerous other databases that contain accurate information focused on interactions among biological entities. WeiBI has an enhanced and enriched pipeline that has been developed by considering all of the existing experimental data in the Gene Expression Omnibus NCBI until March 2019. This huge amount of precise data is being processed and continuously used to improve our proposed database, ensuring the quality of this work.

### Availability and requirements

Server’s home page: http://weislab.com/WeiDOCK/?page=PKPD. Operating system(s): Browser-based (Platform independent). Coding language: Pearl. License: Free License. Restrictions for non-academics: none.

## Supplementary information

Supplementary Table S1.

## Data Availability

The data from and/or analyzed during the current study are available from the corresponding author.
